# Innate Lymphocytes in Adipose Tissue Homeostasis and Their Alterations in Obesity and Colorectal Cancer

**DOI:** 10.3389/fimmu.2018.02556

**Published:** 2018-11-05

**Authors:** Manuela Del Cornò, Lucia Conti, Sandra Gessani

**Affiliations:** Center for Gender-Specific Medicine, Istituto Superiore di Sanità, Rome, Italy

**Keywords:** obesity, colorectal cancer, adipose tissue, immune profile, innate lymphocytes

## Abstract

Colorectal cancer (CRC) is the third most common cancer worldwide and a leading cause of death, with burden expected to increase in the coming years. Enhanced adiposity, particularly visceral fat, is associated with increased cancer incidence representing an important indicator of survival, prognosis, recurrence rates, and response to therapy for several tumors including CRC. Compelling evidence has been achieved that the low-grade chronic inflammation characterizing obesity represents a main factor that can favor carcinogenesis. Adipocytes and adipose tissue (AT) infiltrating immune cells contribute to obesity-related inflammation by releasing soluble factors affecting, both locally and systemically, the function of several cell types, including immune and cancer cells. The unbalanced production of immune mediators as well as the profound changes in the repertoire and activation state of immune cells in AT of obese subjects represent key events in the processes that set the basis for a pro-tumorigenic microenvironment. AT harbors a unique profile of immune cells of different origin that play an important role in tissue homeostasis. Among these, tissue-resident innate lymphocytes are emerging as important AT components whose depletion/aberrant activation occurring in obesity could have an impact on inflammation and immune-surveillance against tumors. However, a direct link between obesity-induced dysfunction and cancer development has not been demonstrated yet. In this review, we provide an overview of human obesity- and CRC-induced alterations of blood and adipose tissue-associated innate lymphocytes, and discuss how the adipose tissue microenvironment in obesity might influence the development of CRC.

## Introduction

White adipose tissue (AT) is a complex immunocompetent organ, enriched with adipocytes and immune cells that contribute to metabolic, endocrine, and immune activities (1). AT is a dynamic organ making up a substantial proportion of the body that, in severe obesity, can account for 50% of body mass (2). This tissue has remained a largely unexplored and unappreciated immune site and only recently emerged that AT harbors a unique profile of immune cells, with either pro-inflammatory (M1 macrophages, dendritic and mast cells, neutrophils, Th1 CD4 and CD8 T cells, B lymphocytes) or anti-inflammatory (M2 macrophages, eosinophils, T_reg_ and Th2 CD4 T cells) activity, playing a key role in immune homeostasis and metabolic regulation of AT (1). Studies carried out in the last decade led to the characterization of non-recirculating lymphocyte populations residing in non-lymphoid tissues and organs such as AT (3). These include unconventional T cells—like invariant natural killer T (iNKT) cells, mucosal-associated invariant T (MAIT) cells, γδ T lymphocytes—the family of innate lymphoid cells (ILC) and tissue-resident memory T cells (3). AT immune cells are key to maintain tissue and immune homeostasis and their profile and function are profoundly altered in obesity (4). The obesity-associated low-grade chronic inflammation represents a major risk factor for related morbidities including some types of cancer. The observation that anti-inflammatory drug use reduces colorectal cancer (CRC) risk and retards intestinal tumors in ulcerative colitis patients has provided compelling evidence for a link among inflammation, obesity, and cancer (5). Growing evidence highlighted the importance of adipocyte microenvironment in modulating the function of immune and cancer cells. The nature of AT/cancer interplay is still largely unknown, but investigations in the field over the last 10 years indicate that this tissue occupies a central place in tumor pathogenesis and progression (6). Systemic and local alterations occurring as a consequence of obesity not only increase the likelihood of tumor development/progression but also set the basis for unfavorable responses to therapy (6).

This review will provide an overview of alterations in blood and AT populations of innate lymphocytes in obesity and CRC. The interplay between AT and cancer cells as a potential mechanism linking obesity to CRC development is discussed.

## Innate lymphocyte profiles in obesity and colorectal cancer

AT exhibits differential profiles in distinct body depots and fat distribution, particularly subcutaneous vs. visceral, has important implications for health (7). The visceral or omental AT (VAT) is immunologically more dynamic than the subcutaneous AT (SAT) and abdominal adiposity is strongly associated with the risk of metabolic disorders, cardiovascular diseases, and cancer (7).

Recent studies clearly showed that the balance between homeostasis and inflammation in AT is mainly controlled by the stromal vascular fraction, that contains, among other immune cells, different populations of innate lymphocytes exhibiting, in homeostatic conditions, regulatory/anti-inflammatory properties (8). Obesity is associated with profound changes in AT infiltrates and systemic profiles of innate lymphocytes, contributing to the dysregulated soluble mediator release and to skewing the balance toward a pro-inflammatory status (8). In addition to act as major regulators of AT homeostasis, innate T cell populations actively contribute to the early defense against cancer (9, 10).

A summary of the main alterations of AT and blood innate lymphocytes in obesity and CRC is provided in Table 1.

**Table 1:** Adipose tissue vs. blood distribution of innate lymphocytes and alterations in their frequencies in obesity and colorectal cancer.

**Cell type**	**Lean**	**Obese**		**CRC**		**References**
	***AT vs. PB***	***AT***	***PB***	***AT***	***PB***
iNKT	AT^∧^> PB					(11, 12)
		↓	↓^*^			(11, 13–17)
		↓	=			(18)
				↓		(11)
NKT-like	VAT > PB					(11, 19)
	VAT = PB					(20)
	VAT > SAT					(21)
		=	=			(11, 19, 20, 22)
				↑	=	(19)
MAIT	VAT > PB	↓	↓↑^#^			(23)
		=				(15)
					↓	(24, 25)
γδ T	VAT = PB					(19)
		=	=	=	=	(15, 19, 26)
Vγ9Vδ2 T			↓			(27)
Vγ9Vδ2/γδ T			↓		↓	(19)
ILC1 (NK)		=	=	=	=	(19, 28)
			↓			(29)
CD56^br^	AT^∧^> PB
CD56^dim^	PB > AT^∧^					(19)
CD56^br^	VAT = PB
CD56^dim^	VAT = PB					(20)
CD56^br^		↑				(30)
ILC2		↓				(31)

PB, peripheral blood; VAT, visceral AT; SAT, subcutaneous AT;

* in both adult and childhood obesity;

# opposite trend in childhood vs. adult obesity;

∧ in both VAT and SAT; =,

↑, ↓: similar, higher or lower frequency as compared to healthy lean controls;

>: *greater than in the comparison between AT and PB*.

### Natural killer T cells

Among innate lymphocytes populating AT are Natural Killer T (NKT) cells, evolutionarily conserved lipid-sensing T cells that strongly influence inflammatory responses (32). They include iNKT, non-invariant NKT and NKT-like cells with the lipid antigen-reactive iNKT cells making up a large proportion (about 10%) of total AT lymphocytes (11, 13, 14, 33). The frequency of iNKT cells in VAT is higher than in any other organ, likely due to lipid antigen abundance in this body compartment and to the antigen presentation capacity of AT-resident myeloid cells including adipocytes (11, 13, 14, 33). Notably, iNKT cell enrichment results from blood recruitment (12) and their frequency correlates with the number of cells expressing the MHC-like glycoprotein CD1d (11).

AT-associated iNKT cells are unusually poised to readily produce anti-inflammatory/regulatory cytokines. They acquire a Th2-like phenotype in response to lipid antigens and have the ability to produce IL-10. Invariant NKT cells also act as regulators of other immune cells thus bridging innate to adaptive immunity (2, 34). In obesity, a reduced iNKT cell frequency was observed in both blood and VAT (11, 14, 15), and in the latter, it parallels a decreased CD1d-expressing cell number (11, 14). Furthermore, iNKT cell frequency inversely correlates with obesity degree, and with insulin resistance and fasting glucose, suggesting that AT resident iNKT cells exert a regulatory role against obesity-associated metabolic disorders (13, 14). Circulating iNKT cell frequency decreases in adult and childhood obesity (16, 17), although other investigators described a reduction only in VAT of adult obese subjects (18). This reduction parallels an increased proportion of early activated iNKT cells in the circulation (18). Interestingly, weight loss induced by gastric bypass surgery in severely obese subjects partially rescues circulating iNKT cell numbers (35) and reverts their activation state (18).

As iNKT cells play a key role in the regulation of inflammation and in cancer surveillance, their contraction in obesity could have important implications for the control of tumor growth. Notably, iNKT cell frequency and CD1d-expressing cell number decline in VAT from CRC affected patients (11).

Like iNKT lymphocytes, the CD1d-independent NKT-like cells are poorly represented in blood of lean individuals and preferentially accumulate in VAT rather than in SAT (11, 19, 21). However, no differences in VAT vs. blood distribution were found in other studies (20). Although not modified in obese subjects (11, 19, 20, 22), these cells are selectively enriched in VAT from CRC patients as compared to healthy individuals, irrespective of BMI (19).

### Mucosal-associated invariant T cells

MAIT cells are emerging as important players in chronic inflammatory disorders including obesity. They express an invariant Vα7.2 TCR chain and high levels of the NK cell-associated receptor CD161, recognize bacterial-derived metabolites presented by the MHC-like molecule MR-1 and are mainly involved in the control of bacterial infections (36). MAIT cells are relatively abundant in the blood and are present in both VAT and SAT (15, 23). Like iNKT cells, they selectively accumulate in lean individual AT, where, unlike their blood counterpart, they produce IL-10 upon stimulation, thus contributing to tissue homeostasis (23).

Obesity profoundly affects MAIT cell number and function. A decreased frequency of circulating MAIT cells was reported in obese individuals. The reduction was found to be stronger in subjects with insulin resistance or with severe obesity, and was counteracted by surgery-induced weight loss (15). In parallel, a preferential activation of MAIT cells, displaying high proliferation and a striking IL-17 profile, was observed in VAT from obese individuals (15). Carolan and co-authors confirmed obesity-related depletion of circulating MAIT cells, but reported a reduced accumulation in VAT (23). An opposite situation was found in obese children, whose circulating MAIT cell frequency is increased and positively correlates with BMI. In both adults and children, however, obesity results in a skewed differentiation of MAIT cells toward an IL-17^+^ phenotype (23).

The role of MAIT cells in cancer is still under debate. Whether they promote malignancy through the production of IL-17, or contribute to anticancer immunity by expressing cytotoxic effector molecules still needs to be elucidated (36). Although alterations of MAIT cell distribution and function have never been studied in the AT of cancer patients, evidence has been provided that the percentages and absolute numbers of circulating MAIT cells decrease in gastric and colon tumors (24, 25). This depletion only occurs in patients affected by mucosal-associated cancers and is associated with an increased number of tumor infiltrating MAIT cells, especially in subjects with advanced CRC, thus reflecting the degree of cancer progression (24, 25).

### γδ T lymphocytes

γδ T cells sense self and non-self danger signals expressed in stressed, infected or tumor cells by recognizing non-peptidic phosphorylated antigens in a MHC-independent manner (37). They exert their effector functions either directly by cytotoxic activity or indirectly through the production of immune mediators and activation of other immune cell populations (37). In humans, three major subsets have been identified: Vδ1 T cells, predominantly present in the thymus and peripheral tissues; Vδ2 T cells, always expressing the Vγ9 chain, which constitute the majority of blood γδ T lymphocytes and can be recruited in inflamed tissues in pathologic conditions (38); Vδ3 T cells, poorly represented in the blood and enriched in the liver.

Despite studies demonstrating a role of IL-17-producing γδ T cells in murine obesity, the involvement of γδ T cells in human obesity-associated chronic inflammation has been only poorly investigated. We recently reported comparable frequencies of blood and VAT-associated total γδ T lymphocytes in obese and CRC-affected subjects as compared to healthy lean individuals (19). Accordingly, no alterations in circulating γδ T cells were reported in obese (15) and CRC-affected subjects (26). However, when the analysis was extended to the Vγ9Vδ2 subset, we observed a systemic reduction of the Vγ9Vδ2/γδ T cell ratio, inversely correlating with BMI, in both obese subjects and CRC patients (19). Likewise, a reduced number of blood Vγ9Vδ2 T cells was reported in obese individuals, accompanied by a skewed maturation phenotype and impaired IFN-γ production, mainly attributed to IL-2 deprivation in obesity (27). The low frequency of Vγ9Vδ2 T cells with reduced activation potential in obesity could negatively impact cancer immune-surveillance.

### Innate lymphoid cells

ILC, a heterogeneous immune cell population not expressing T- or B-cell receptor, include ILC1, ILC2, and ILC3 groups, characterized by the ability to produce Th1-, Th2-, and Th17-like cytokines, respectively (39). They contribute to homeostasis in both barrier and non-barrier tissues and have been implicated in infection, chronic inflammation, and metabolic disorders (39). Among ILC, NK cells, belonging to ILC1 group, and ILC2 have been described in human AT where they contribute to inflammation or to maintain tissue homeostasis, respectively.

A selective distribution of NK cell subsets in VAT and blood was recently reported in lean individuals, with CD56^bright^ cells preferentially enriched in VAT and CD56^dim^ cells more represented in the blood (19). Conversely, NK cell subsets were found to be equally distributed in VAT/SAT and blood compartments in previous studies (20). The VAT/blood distribution was not substantially changed in obesity (20, 28), although a slight increase in VAT CD56^bright^ and a parallel decrease in CD56^dim^ NK cell frequency was detected (20). A reduced number of NK cells, associated to an expansion in inhibitory-receptor (CD158b, NKB1) bearing cells, was found in the peripheral blood of obese individuals, particularly in metabolically unhealthy subjects (29). Obese subject circulating NK cells were also reported to express higher levels of activation markers despite an impaired cytotoxic activity against tumor cells (40, 41). Furthermore, a selective accumulation of a distinct IL-6 receptor-expressing subset, correlating with systemic low-grade inflammation markers, was recently reported in the circulation of obese subjects (28). Conversely, Dovio and colleagues failed to detect any obesity-associated NK cell dysfunction (42). In addition, O'Rourke and colleagues reported an activated phenotype also for AT resident NK cells that, in obesity, upregulate the NKG2D receptor (20). Although the total NK cell number is similar in blood and AT of lean and obese subjects, affected or not by CRC, NK cells residing in SAT of obese individuals display a poor cytotoxicity against target tumor cells (19, 28, 30). Moreover, reduced NK cell survival and expansion of IL-10-producing NK cells have been described in other obesity-associated cancers (43). Altogether, these data suggest that NK cell chronic stimulation could occur in obesity leading to their exhaustion. This could contribute to the greater susceptibility of obese individuals to develop cancers or infectious diseases.

ILC2 play an important role in mounting protective innate responses against parasites and helminthes and in maintaining intestinal epithelium integrity (44). Due to their capacity to secrete anti-inflammatory cytokines, they may act as anti-obesity immune regulators. However, despite the evidence obtained in mouse models, studies investigating ILC2 abundance in human AT and their role in metabolic homeostasis are scarce. Cells expressing GATA binding protein 3 and IL-33 receptor, consistent with ILC2 described in other human tissues, have been recently identified in lean individual SAT and their frequency decreases in obese subjects, as already described for murine obesity (31).

## Influence of adipose tissue microenvironment on immune and colorectal cancer cells

### AT modulation of innate lymphocyte function

Growing evidence points to the existence of a cross-talk between AT and immune cells with the latter able to influence adipocyte metabolic function (45). Less is instead known about the influence of AT on cells of the immune system, particularly innate lymphocytes. Although a variety of mediators (cytokine, chemokines, lipids and their metabolites, hormones and growth factors) released by adipocytes have the potential to modulate innate lymphocyte functions and recruitment (46), the studies describing adipocyte/innate T cell interplay failed to characterize the specific factors involved. In particular, evidence was provided that MAIT cell function is influenced by AT released soluble factors. It was reported that the frequency of IL-17-producing MAIT cells increases in the presence of AT conditioned media (CM) from obese subjects, while that of IL-10-producing MAIT cells is increased by CM from lean subjects (23).

Likewise, CM from obese individuals strongly reduce the expression of the survival molecule Bcl-2 while increasing the percentage of Ki-67-expressing MAIT cells (15). These observations suggest that soluble factors released by AT in obesity might lead to the death of MAIT cells or skew their differentiation toward a pathogenic phenotype thus contributing to the establishment/perpetuation of chronic inflammation.

We also provided evidence that, in obesity and CRC conditions, adipocyte microenvironment delivers immunosuppressive signals to differentiating dendritic cells (DC), as assessed by their enhanced expression of inhibitory molecules and reduced IL-12/IL-10 ratio (47). Likewise, DC generated in adipocyte CM from obese and CRC subjects fail to induce the activation of Vγ9Vδ2 T lymphocytes. Of note, adipocytes from obese and CRC subjects release higher amounts of pro-inflammatory/immunoregulatory cytokines/chemokines (IL-6, CXCL8, CCL2), and exhibit a higher content of pro-inflammatory ω6 polyunsaturated fatty acids (FA), with respect to lean subjects (47).

An intimate interaction between adipocytes and iNKT cells has been reported in mouse models, where CD1d-expressing adipocytes contribute to lipid antigen presentation and activation of iNKT cells (14). Although an interaction between adipocytes and iNKT cells has not been demonstrated yet in human AT, the correlation between CD1d expression and iNKT cell frequency in lean subject AT, and their concomitant reduction in obesity (11, 13, 14, 33), point to the existence of a dynamic cross-talk between these cell populations. Depending on the lipid antigens produced in response to nutritional environment, iNKT cells might be primed toward either anti- or pro-inflammatory phenotypes, thus contributing to AT homeostasis or inflammation, respectively. In this regard, it is of interest that adipocyte secreted lipids are potent inhibitors of LPS-induced IL-12p40 secretion in macrophages. This effect is AT depot-independent and its extent correlates with BMI (48). Likewise, adipocyte-derived lipids, particularly free FA, exert a potent immunomodulatory effect by stimulating CD4^+^ T cell proliferation in an AT depot-independent manner (49).

All together these observations provide evidence for a cross-talk between AT and innate T cells, at least in part promoted by soluble mediators (lipids or cytokines/chemokines or still unknown factors), that is dysregulated in obese and cancer affected subjects thus leading to altered tissue recruitment and function of innate immunity cell subsets.

### AT cross-talk with colorectal cancer cells

The altered systemic and AT environment occurring in obesity not only increases the likelihood of tumor development/progression but also sets the basis for unfavorable responses to therapeutic regimens (9, 10). Several studies suggest that, within the constitutively active pro-inflammatory AT microenvironment characterizing obesity, the bidirectional cross-talk between AT and cancer cells may play an important role (50). Such interplay occurs via deregulated expression of mediators of different nature: cytokines (i.e., IL-1, IL-6, TNFα), chemokines (i.e., CCL2, CXCL8), lipids and their metabolites (i.e., free FA, PGE_2_), hormones (estrogens), growth factors (i.e., IGF-1, VEGF), that are potentially linked to increased risk of cancer development/progression (21, 47–49, 51, 52). Growing evidence suggests that non-cancer cell types in the tumor microenvironment, such as adipocytes and infiltrating immune cells, interact to enhance inflammation, reprogram cancer cell metabolism, and affect processes involved in invasion, metastasis, and immune clearance, all of which can support tumor progression and impact patient outcome (53).

In this regard, Amor et al. (51) reported enhanced expression of pro-inflammatory and angiogenic factors (i.e., nitrite/nitrate, IL-6, TNF-α, VEGF) by peritumoral AT of CRC affected obese patients with respect to lean subjects. Likewise, Catalan and colleagues showed increased release of pro-inflammatory and angiogenesis-related factors (lipocalin-2, chitinase 3-like 1, TNF-α, osteopontin, HIF-1α, VEGF), as well as increased MMP9 enzymatic activity in VAT of overweight subjects affected by CRC (52). On the other side, lipoprotein lipase and FA synthase activity were found to be reduced in peritumoral AT suggesting a tumor-induced impairment of lipid storing capacity of adipocytes in CRC patients (54).

Only few studies investigated the direct interaction between AT and cancer cells in *in vitro* models. Lysaght and colleagues reported higher levels of VEGF in serum and VAT CM from centrally obese as compared to non-obese CRC patients (21). Interestingly, CM from obese subjects increase the proliferation of colon cancer cells in a VEGF-mediated manner. By comparing SAT and VAT depots, the latter was shown to contain higher levels of VEGF and IL-6 (21). Furthermore, adipocyte secreted factors from obese subjects decrease oxygen consumption rate and expression of mitochondrial respiratory chain complexes in colon cancer cells. The observed inhibition of mitochondrial respiration and function is characteristic of the metabolic reprogramming of malignant transformation and is partially mediated by leptin (55).

Evidence on the existence of a direct relationship between AT-associated innate lymphocyte dysfunctions and CRC development is still lacking. We propose that the contribution of innate lymphocytes to obesity-associated tumor development/progression relies on: (i) alteration of cytokine profile that contributes to inflammation and ultimately affects intestinal mucosa homeostasis, and (ii) impaired direct antitumor activity that favors a shift from immune-surveillance toward tumor escape.

## Innate lymphocyte alterations in obesity and their impact on CRC

Obesity is associated with profound changes in AT and systemic profiles of innate lymphocytes, contributing to dysregulated soluble mediator release and to skewing the balance toward a pro-inflammatory status. In addition, the cytotoxic activity of innate lymphocyte populations and their capacity to directly orchestrate the early defense against cancer are impaired in obese individuals.

A number of studies have documented the critical role of these cells in tumor immunity and highlighted the therapeutic potential of their targeting in different cancer types including CRC (9, 10). Activated innate lymphocytes produce cytokines and cytotoxic factors and are capable of directly lysing tumors. Furthermore, they can drive the activation of other immune cell populations, such as DC, and are able to target tumor-associated macrophages, myeloid-derived suppressor cells and IL-10-secreting neutrophils which generally characterize the immunosuppressive tumor microenvironment, as well as cancer stem cells (9, 10). Thus, alterations of AT and blood innate lymphocyte frequencies and functions occurring in obesity are expected to have an impact on both inflammation and surveillance against tumors. In particular, MAIT cells have the potential to either promote malignancy through the production of IL-17, or contribute to anticancer immunity by expressing cytotoxic effector molecules (36). The skewed MAIT cell differentiation toward an IL-17 producing phenotype occurring in obese subjects could at least in part explain the detrimental role of AT inflammation in the generation of a pro-tumorigenic environment. Of note, aberrant activation of the IL-23/IL-17 axis has been linked to tumorigenesis and adverse prognosis in CRC (56), suggesting that the hyper-production of IL-17 occurring in AT could be of relevance for obesity-associated CRC pathogenesis. Likewise, the exhausted phenotype of NK cells, the aberrant activation of iNKT cells and the contraction of the Vγ9Vδ2 T lymphocyte subset observed in obese subjects could contribute to the greater susceptibility of these individuals to develop cancer or infectious diseases. Indeed subjects with low circulating NK cell activity have a 10-fold higher risk of developing CRC compared to subjects with high NK cell activity, and the HNK1 NKG2D genotype, associated with high NK cell activity, exerts a protective effect against CRC (57, 58). In support of this, alterations of some of these innate lymphocyte subsets have also been observed in blood or AT of CRC patients, suggesting a direct link between obesity-induced dysfunctions and cancer.

## Concluding remarks

**Figure 1:**
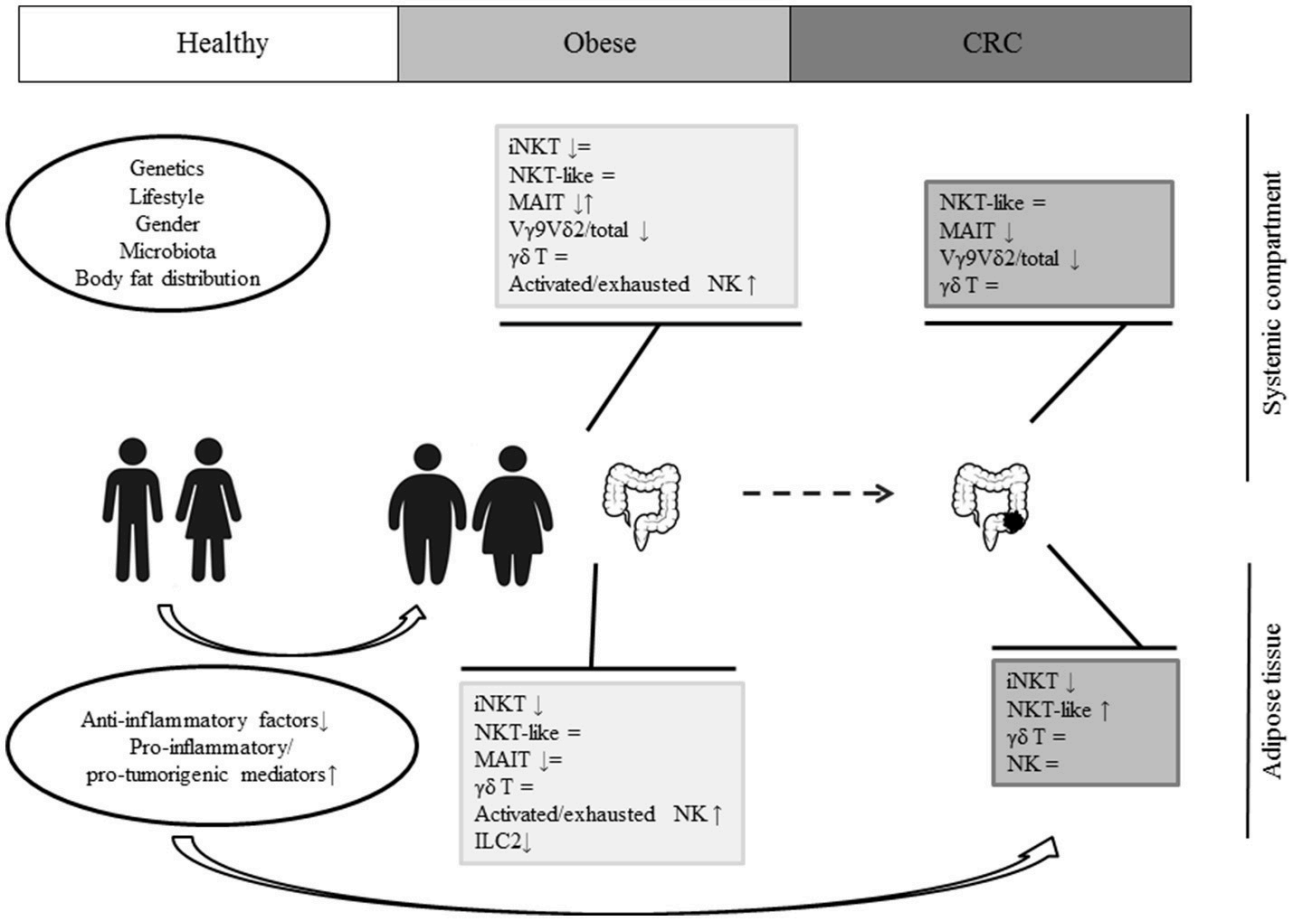
A schematic representation of innate lymphocyte dysfunctions in obesity and CRC.

Obesity results from a complex interplay of different factors including genetics, gender, lifestyle, microbiota, and body fat distribution. The shift from a lean to an obese condition is associated with profound changes in the balance between anti- and pro-inflammatory mediators, release of pro-tumorigenic factors and alteration of local and systemic immune profiles. As schematically depicted in Figure 1, a selective obesity-associated depletion and/or reduced activity of different innate lymphocyte populations has been recently highlighted, pointing to a key role of these cells in the control of inflammation. However, although it is likely that obesity-associated inflamed/immunosuppressive environment can ultimately alter intestinal mucosa homeostasis and support tumor development/progression, the role of innate lymphocytes in this process has not been fully elucidated yet. Moreover, whether their capacity to home to the tumor site and to perform their effector functions is impaired in obesity remains to be defined. If this knowledge is acquired, it will provide further rationale to the promising approach of boosting the effector functions of innate lymphocytes, in addition to those of conventional T cells, to improve cancer immunotherapy.

Obesity is a major risk factor for different cancer types, including CRC, with the latter known to be highly modifiable through lifestyle. Further studies aimed at dissecting the influence of a healthy/unhealthy lifestyle on the mechanisms linking obesity to CRC will allow a better exploitation of lifestyle-based interventions in controlling tissue homeostasis and immune-surveillance.

## Author contributions

MD, LC, and SG contributed to the conception, writing, and editing of this manuscript. All authors read and approved the final manuscript.

### Conflict of interest statement

The authors declare that the research was conducted in the absence of any commercial or financial relationships that could be construed as a potential conflict of interest.
